# Ultra-sensitive *in situ* detection of intracellular Mycobacterium tuberculosis with CRISPR/Cas12a

**DOI:** 10.3389/fimmu.2025.1597654

**Published:** 2025-05-21

**Authors:** Zhiqiang Lin, Zhe Song, Hong Yu, Yang Zhou, Deliang Liu, Peize Zhang, Li Wei, Guiqin Dai, Guangyan Liang, Zhuojun He, Xiaorong Hu, Yuying Chen, Pengfei Zhao, Hongzhou Lu, Mingbin Zheng

**Affiliations:** ^1^ National Clinical Research Center for Infectious Disease, Shenzhen Third People’s Hospital, Southern University of Science and Technology, Shenzhen, China; ^2^ Molecular Biology Research Center & Center for Medical Genetics, School of Life Sciences, Central South University, Changsha, China; ^3^ Department of Respiratory and Critical Care Medicine, Linyi People’s Hospital, Linyi, China; ^4^ The Affiliated Dongguan Songshan Lake Central Hospital, Guangdong Medical University, Dongguan, China

**Keywords:** mycobacterium tuberculosis, CRISPR/Cas12a, macrophage, intracellular detection, bronchoalveolar lavage fluid

## Abstract

*Mycobacterium tuberculosis* (*Mtb*) invades and survives inside macrophages, evading detection and resisting antibiotic treatment, which results in severe clinical consequences such as fatal respiratory failure and systemic inflammation. Rapid and specific detection of intracellular *Mtb* is crucial for accurate diagnosis and optimizing treatment strategies. In this study, we developed a one-step CRISPR/Cas12a assay targeting the *IS6110* gene for the specific and rapid detection of intracellular *Mtb*. Upon efficient delivery into RAW264.7 macrophages, the assay enabled direct visualization of *Mtb IS6110* nucleic acid, generating detectable fluorescence signals. The diagnostic performance was further validated using bronchoalveolar lavage fluid (BALF) samples from clinical participants, achieving a sensitivity of 94%, which surpassed conventional methods such as culture (67%) and Xpert (78%), while maintaining a specificity of 100%. This CRISPR/Cas12a-based assay offers a highly sensitive, rapid, and innovative approach for intracellular *Mtb* detection, with significant potential to enhance tuberculosis diagnostic methodologies and improve clinical outcomes.

## 1 Introduction

Tuberculosis (TB) remains one of the deadliest infectious diseases worldwide (1). Its causative pathogen, *Mycobacterium tuberculosis* (*Mtb*), survives within macrophages by evading lysosomal degradation and regulating host metabolic pathways to establish a protective intracellular microenvironment (2). This strategy not only protects *Mtb* from antimicrobial drugs but also promotes antibiotic resistance, thereby increasing transmission risk. Persistent intracellular *Mtb* is linked to extrapulmonary TB, chronic recurrent infections, and multidrug-resistant tuberculosis (MDR-TB) (3). Thus, a highly sensitive, specific, and rapid method for *in situ* detection of intracellular *Mtb* is essential for optimizing treatment and controlling disease spread.

Current methods for detecting intracellular *Mtb* include bacterial culture, Acid-Fast Bacilli (AFB) staining, molecular diagnostics (e.g., the GeneXpert MTB/RIF assay (Xpert), Tuberculosis DNA detection (TB-DNA), and whole-genome sequencing), and immunological tests such as tuberculin skin tests and γ-interferon release assays (4). Although these techniques provide certain advantages, they also possess notable limitations. *Mtb* culture (5) requires prolonged incubation (up to three weeks), delaying timely clinical intervention. AFB staining (6), despite its widespread use, suffers from low sensitivity and high false-negative rates. PCR-based detection (7) is vulnerable to aerosol contamination, which can compromise reproducibility and accuracy. Moreover, these methods necessitate cell fixation or lysis, limiting their application in detecting *Mtb* in living cells. Thus, there is an urgent need for a simple and specific strategy to detect intracellular *Mtb* in real time.

The CRISPR/Cas (Clustered Regularly Interspaced Short Palindromic Repeats) system has emerged as a powerful tool for intracellular nucleic acid detection, providing both high accuracy and a visual readout (8, 9). Among these systems, CRISPR/Cas12 shows particular promise in bacterial diagnostics owing to its sensitivity, specificity, rapid detection, and multiplexing capabilities (10–13). Cas12 recognizes double-stranded DNA (dsDNA) and, when activated by a T-rich protospacer adjacent motif (PAM), induces collateral cleavage of single-stranded DNA (ssDNA). The system employs a fluorescence-quenched (F-Q) ssDNA probe to suppress background noise and generate a strong fluorescent signal upon target recognition. For instance, Peng et al. combined CRISPR/Cas12b with cross-priming amplification (CPA) to detect *Mtb* in sputum samples, achieving sensitivity of 67.2% and specificity of 96.7% in clinical evaluation (14). These findings underscore the potential of CRISPR/Cas12 system for rapid and sensitive *Mtb* detection.

Here, we report a one-step CRISPR/Cas12a assay for the *in situ* detection of intracellular *Mtb* nucleic acids in living macrophages. Cas12a, guided by crRNA, targets the *IS6110* sequence (15–17) and triggers the cleavage of an F-Q probe, yielding a quantifiable fluorescence signal. By optimizing the ribonucleoprotein (RNP) complexes and fluorescent probes, our assay enables real-time visualization of *Mtb* nucleic acids *via* confocal microscopy. Validation with bronchoalveolar lavage fluid (BALF) samples from clinical participants demonstrated high sensitivity and specificity, highlighting its potential for clinical application. This study introduces CRISPR/Cas12a as an ultra-sensitive and specific diagnostic tool for detecting intracellular *Mtb*, representing a significant advancement in TB diagnostics, particularly for cases with low bacterial loads.

## 2 Materials and methods

### 2.1 Materials and reagents

LbCas12a recombinant protein (Cas12a) and HOLMES Buffer were purchased from Shanghai Tolo Biotech Co., Ltd (CHN). The primers, crRNA and F-Q probe were chemically synthesized by Shanghai Sangon Biotechnology Co., Ltd (CHN). The electroporation cuvettes (0.4cm) were purchased from Bio-Rad Laboratories (USA). Difco Middlebrook 7H9 were purchased from Becton Dickinson (USA). Dulbecco’s modified Eagle’s medium (DMEM), phosphate buffered saline (PBS), penicillin, streptomycin, and fetal bovine serum (FBS) were purchased from Gibco (Thermo Fisher Scientific, USA). Hoechst 33342 was bought from Beyotime Biotechnology (CHN). All aqueous solutions were prepared using ultrapure water.

### 2.2 Preparation of oligos and crRNA

Primers were designed and synthesized for the amplification of the *IS6110* region, including the forward primer 5’-GGTCGGAAGCTCCTATGACAATGCACTAGCC-3’ and the reverse primer 5’-TTGAGCGTAGTAGGCAGCCTCGAGTTCGAC-3’. In this study, a crRNA with the sequence of 5’-UAAUUUCUACUCUUGUAGAU AUCAGCUCG GUCUUGUAUAG-3’ was employed, which is proximal to the PAM sequence (5’-TTTG-3’). Additionally, a F-Q probe (5’-6-FAM-TTTTTTTTTTTT-BHQ-1) was utilized. The *IS6110* plasmid (10^5^ copies/μL) was a gift from Prof. Zhen Huang’s lab. For the optimization of the CRISPR/Cas12a assay, the amplification product of *IS6110* plasmid served as the reaction substrate and positive control.

### 2.3 Bacterial strains and cells

*Mycobacterium tuberculosis H37Ra* (ATCC25177), *BCG* (35737) and RAW264.7 macrophage cells were purchased from the American Type Culture Collection (ATCC, USA). Other species of nontuberculous mycobacteria (NTM), including, *M. smegmatis*, *M. kansasii*, *M. abscessus*, *M. avium*, and two species of bacteria, *E. coli* and *S. aureus* were purchased from China General Microbiological Culture Collection Center (CGMCC). *H37Ra*, *BCG*, *M. smegmatis*, *M. kansasii*, *M. abscessus* and *M. avium* were cultured in Middlebrook 7H9 liquid medium (50 mL) supplemented with 10% oleic albumin dextrose catalase (Biorab, USA) and 0.5% glycerol (Sigma-Aldrich, USA). *E. coli* and *S. aureus* were cultured in LB medium (Solarbio, CHN). All cultures were shaken at 100 rpm (37°C). For labeling, *H37Ra* was incubated with 0.4 µL TPAPy-D-Ala (synthesized in our lab) for 2 h (37°C), centrifuged (12,000 rpm, 5 min), washed thrice with PBS, and resuspended in DMEM.

RAW264.7 macrophages were maintained in DMEM with 10% FBS and antibiotics (100 U/mL penicillin, 100 µg/mL streptomycin) at 37°C/5% CO₂. Cells (2×10⁵ cells/mL) were infected with *H37Ra* at a multiplicity of infection (MOI) of 1:10 for 4 h, washed with PBS, detached using 0.25% Trypsin-EDTA (Gibco, CHN; 1 min, 37°C), centrifuged, and resuspended in 300 µL Opti-MEM (Gibco, CHN) for downstream use.

### 2.4 Investigating the detection performance of CRISPR/Cas12a system

The presence of crRNA-Cy3 in the cells was assessed by flow cytometry. Following electroporation, the cells were centrifuged, resuspended in PBS, and analyzed using a BD FACSymphony™ A3 flow cytometer (BD Biosciences, USA). The presence of Cas12a in the cells was analyzed by western blot. Briefly, cells were lysed in RIPA buffer (Thermo Fisher, USA) with 1% protease (Beyotime, CHN) and phosphatase inhibitors (Beyotime, CHN). Protein concentration was measured using Bradford Reagent (Thermo Fisher, USA). After blocking (5% milk in PBS, 1 h), membranes were incubated with a primary antibody (Anti-LbCas12a, Sigma-Aldrich, USA) overnight at 4°C, followed by a secondary antibody (Abcam, UK) for 2 h at room temperature. Protein bands were imaged to confirm Cas12a presence.

The *IS6110* plasmid PCR product (10⁸ copies/μL) was used as the substrate for optimizing F-Q probe and Cas12a RNP concentrations. The RNP complex was assembled by incubating crRNA and LbCas12a (10 μM in HOLMES Buffer) at a 1:1 (*v/v*) ratio in DEPC-treated water at 20-25°C for 15 min. For optimization, 5 μL of *IS6110* DNA was added to 25 μL reaction mixtures containing different concentration of F-Q probe (0.5 µM, 1 µM, 1.5 µM, 2 µM) or Cas12a RNP (6.25 nM, 12.5 nM, 25 nM, 50 nM, 100 nM). Fluorescence intensity was measured using a Varioskan Lux microplate reader (USA) within 2 hours. Specificity was evaluated using *IS6110* DNA as a positive control and RNase-free water as a negative control. 5 μL genomic DNA from various bacterial strains was used in the test.

### 2.5 Detecting intracellular *Mtb* using CRISPR/Cas12a

The F-Q probe (100 μM) was added with Cas12a RNP (10 μM in HOLMES Buffer), yielding final concentrations of 50 nM crRNA, 50 nM LbCas12a, and 1500 nM F-Q probe. For cell internalization, 100 μL of the RNP complex solution was mixed with 300 μL of macrophage suspension (10⁷ cells/mL) infected with *H37Ra* and transferred to a 4 mm gap cuvette. Electroporation was performed using a Gene Pulser Xcell (Bio-Rad, USA) (Square wave, 250V, 10 ms). Cells were centrifuged, washed with PBS, resuspended in 100 μL DMEM, and incubated at 37°C for fluorescence monitoring over 2 hours. Signals (Excitation at 488 nm; Emission at 525 nm) were detected *via* a microplate reader (Varioskan Lux, USA) or imaged using Zeiss LSM 980 confocal microscope (Carl Zeiss Inc. Germany).

### 2.6 Participants recruitment, sample collection and intracellular Mtb detection

This study adhered to the ethical principles outlined in the Declaration of Helsinki. Patient recruitment and the collection of BALF samples were approved by the Research Ethics Committee of Shenzhen Third People’s Hospital (approval number: 2021-030). Written informed consent was obtained from all participants for sample collection and subsequent analyses. The clinical diagnosis of TB was based on a combination of clinical symptoms, chest radiography findings, microscopy for acid-fast bacilli (AFB), *Mtb* culturing, Xpert analysis of sputum and/or BALF, and response to anti-TB chemotherapy. A total of 28 BALF samples were collected, comprising 10 samples from healthy donors and 18 *Mtb*-positive samples from tuberculosis patients.

For the detection of clinical samples, 3 mL of BALF was filtered (70 µm), centrifuged (1000 rpm, 5 min), and resuspended in DMEM. Alveolar macrophages, the predominant cells, were electroporated with the CRISPR/Cas12a assay, and fluorescence was measured within 2 hours using a microplate reader.

### 2.7 Statistical analysis

All experimental data were presented as means ± standard error of mean (SEM), with experiments repeated at least three times. A Mann-Whitney U test was used for comparisons between the two groups. In all tests, *P* values < 0.05 were considered statistically significant. *** indicated *P* < 0.001. The statistical analysis was performed using GraphPad Prism 8.0 software.

## 3 Results and discussion

### 3.1 Schematic diagram and optimization of CRISPR/Cas12a assay

**Figure 1:**
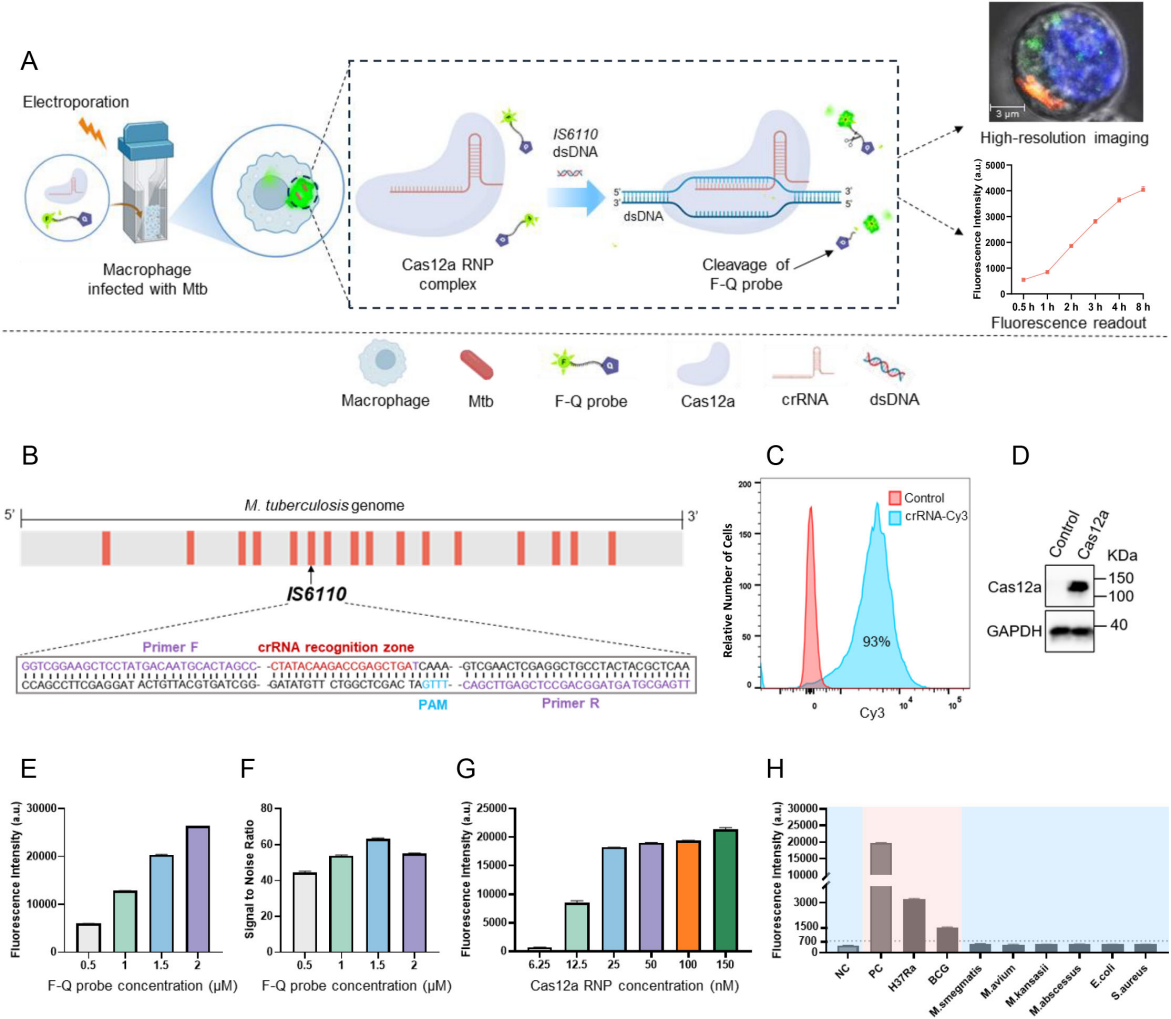
Schematic diagram and optimization of CRISPR/Cas12a assay. **(A)** Schematic illustration of the CRISPR/Cas12a assay for intracellular *Mtb* detection. Cas12a-crRNA RNP complexes were introduced into *H37Ra*-infected RAW264.7 macrophages *via* electroporation. Guided by crRNA, the Cas12a protein specifically recognized the *IS6110* target sequence, triggering the cleavage of F-Q probe, which generated measurable fluorescence signals in macrophages for high-resolution imaging or detection. **(B)** The CRISPR/Cas12a target sequences were mapped in the genome of *Mycobacterium tuberculosis* (*H37Ra*). **(C)** The successful introduction of Cy3-labeled crRNA targeting *IS6110* into macrophages *via* electroporation was validated using flow cytometry. **(D)** The presence of Cas12a protein in macrophages was determined using western blotting. **(E)** The fluorescence intensity generated by F-Q probe at varying concentrations. **(F)** The signal-to-noise ratio (SNR) varied with the concentration of the F-Q probe. **(G)** The fluorescence intensity generated by Cas12a RNP complexes at varying concentrations (n = 3, means ± SEM). **(H)** DNA of *H37Ra*, *BCG* strain and 6 NTM strains were subjected to CRISPR/Cas12a assay to determine the detection specificity (n = 3, means ± SEM).

Herein, we developed a CRISPR/Cas12a-based diagnostic platform for the rapid, ultra-sensitive, and highly specific detection of intracellular *Mtb*. The platform employed Cas12a crRNA ribonucleoprotein (RNP) complexes and a quenched fluorescence (F-Q) probe, which were delivered into RAW264.7 macrophages infected with the *Mtb H37Ra* strain *via* electroporation (**Figure 1A**). Once inside the cells, the crRNA guided the Cas12a protein to specifically recognize the *IS6110* target sequence, triggering the trans-cleavage of the F-Q probe. This cleavage generated fluorescence signals, which could be visualized through confocal microscopy or quantified using a microplate reader. To enhance molecular sensitivity, the *IS6110* insertion sequence-unique to *Mtb* and present in multiple copies per genome-was selected as the detection target. The primer and crRNA sets targeting different regions of *IS6110* were shown in **Figure 1B** and **Table 1**. To evaluate the efficiency of electroporation for delivering the assay components, the crRNA was labeled with a Cy3 fluorescent marker and analyzed *via* flow cytometry. The results showed that 93% of macrophages successfully incorporated the labeled crRNA (**Figure 1C**). Additionally, western blot analysis of lysed cells confirmed the presence of Cas12a protein in the macrophages (**Figure 1D**). Together, these findings verified the successful delivery of both crRNA and Cas12a into the host cells, establishing a solid foundation for the functionality of the diagnostic platform.

**Table 1:** Oligonucleotide sequences (5’-3’) used in this study.

Name	Sequences (5′-3′)	Length
*IS6110-*F	GGTCGGAAGCTCCTATGACA ATGCACTAGCC	31nt
*IS6110-*R	TTGAGCGTAGTAGGCAGCC TCGAGTTCGAC	30nt
crRNA-*IS6110*	UAAUUUCUACUCUUGUAGA UAUCAGCUCGGUCUUGUAUAG	40nt
F-Q probe	5′-FAM-TTTTTTTTTTTT-BHQ-3′	12nt

Underlined sequence indicates the region complementary to the *IS6110* target sequence.

To enhance efficiency and accelerate the reaction, two key components of the assay system (F-Q probe and Cas12a RNP complexes) were optimized by adjusting one reagent at a time in each experiment. It is worth noting that the amplification product of *IS6110* DNA served as the reaction substrate in this optimization experiment. The results demonstrated a progressive increase in fluorescence intensity as the probe concentration was increased from 0.5 µM to 2 µM. Notably, the system achieved the optimal signal-to-noise ratio at a probe concentration of 1.5 µM (**Figures 1E, F**). Furthermore, with the probe concentration fixed at 1.5 µM, a distinct increase in fluorescence intensity was observed as the RNP complexes concentration was raised from 6.25 nM to 150 nM. The results showed that fluorescence intensity plateaued at a Cas12a RNP complexes concentration of 50 nM, with only minimal increases observed at 100 nM and 150 nM. Thus, 50 nM was identified as the optimal concentration for Cas12a RNP complexes (**Figure 1G**). Finally, the assay’s specificity was evaluated using a panel of bacterial strains, including two attenuated *Mtb* strains (*H37Ra* and *BCG*), four non-*Mtb* strains (*M. smegmatis*, *M. kansasii*, *M. abscessus*, and *M. avium*), and two Gram-negative bacteria (*E. coli* and *S. aureus*). Significant fluorescence signals were detected exclusively in *Mtb* strains, while the signals generated by the DNA of non-*Mtb* and Gram-negative bacteria were comparable to background levels. These results confirmed the high sensitivity and specificity of the CRISPR/Cas12a assay for *Mtb* detection (**Figure 1H**).

### 3.2 Evaluation of the CRISPR/Cas12a assay for detecting intracellular *Mtb* and clinical samples

**Figure 2:**
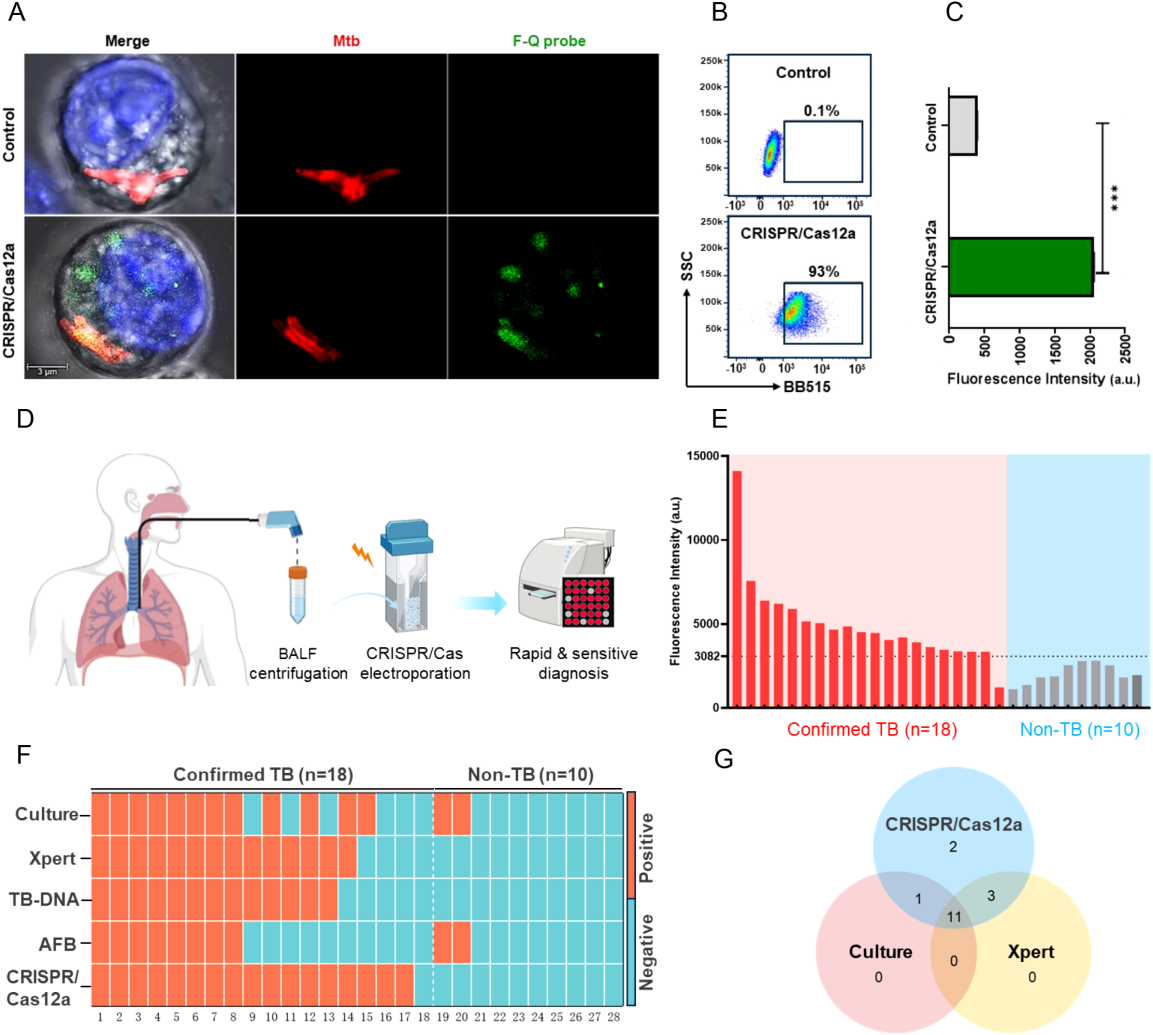
Evaluation of the CRISPR/Cas12a assay for detecting intracellular *Mtb* and clinical samples. **(A)** Confocal microscopy images of RAW264.7 macrophages. Nuclei (blue) and *Mtb* (red) were labeled, while the F-Q probe emitted green fluorescence upon activation. In the CRISPR/Cas12a group, partial co-localization of green fluorescence with *Mtb* appeared yellow in the merged image. **(B)** Flow cytometry results showed the percentage of fluorescence-positive cells across groups. **(C)** Median fluorescence intensity of F-Q probe-positive cells analyzed by flow cytometry (Mann-Whitney U test, ***P < 0.001, n = 3, means ± SEM). **(D)** Workflow of the CRISPR/Cas12a assay for clinical sample testing. BALF samples were filtered, centrifuged, and resuspended in DMEM before electroporation with the CRISPR/Cas12a assay. Fluorescence was measured using a microplate reader within 2 hours. **(E)** Blind testing of 28 BALF samples (18 TB-positive, 10 non-TB) using the CRISPR/Cas12a assay. **(F)** Heatmap summarizing diagnostic results of BALF samples across five methods: Culture, Xpert, TB-DNA, AFB, and CRISPR/Cas12a. **(G)** Venn diagram showing overlap in *Mtb*-positive detections among CRISPR/Cas12a, Culture, and Xpert.

To evaluate the diagnostic potential of Cas12a RNP complexes for detecting *Mtb* in macrophages, *IS6110* was selected as the target gene. Cas12a RNP complexes were electroporated into RAW264.7 macrophages infected with *H37Ra*, and fluorescence signals were visualized *via* confocal microscopy after 2 hours. *H37Ra* bacteria were pre-labeled with TPAPy-D-Ala aggregation-induced luminescence probes (red), while macrophage nuclei were stained with Hoechst 33342 (blue). Red fluorescent bacteria predominantly localized in the cytoplasm. In the CRISPR/Cas12a group, green signals corresponding to *IS6110* overlapped with red bacterial signals, forming bright yellow areas, while isolated green signals appeared elsewhere in the cytoplasm (**Figure 2A**). This suggests that while some *Mtb* survived phagocytosis, others were lysed, releasing nucleic acids into the cytoplasm (18, 19). Additionally, viable *Mtb* may have secreted nanovesicles carrying nucleic acids, including *IS6110* DNA (20–22). Upon binding of *IS6110* DNA to the Cas12a RNP complexes, the trans-cleavage activity of Cas12a was triggered, resulting in the cleavage of the F-Q probe and the production of a green fluorescent signal. Thus, the yellow signals likely represented nucleic acids associated with intact bacteria, while the isolated green signals reflected dispersed nucleic acids from lysed bacteria or secreted nanovesicles. Flow cytometry revealed fluorescent signals in 93% of macrophages, significantly exceeding the control (**Figure 2B**), with median fluorescence intensity approximately fourfold higher (**Figure 2C**). These results demonstrate that the CRISPR/Cas12a assay enabled real-time, *in situ* imaging of *Mtb* nucleic acids within macrophages, demonstrating its potential as a powerful diagnostic tool for detecting low-abundance intracellular *Mtb*.

Furthermore, to evaluate the clinical performance of the CRISPR/Cas12a assay, 18 bronchoalveolar lavage fluid (BALF) samples from patients with active pulmonary tuberculosis (TB) and 10 BALF samples from patients with non-TB respiratory infections were analyzed. *IS6110* DNA served as the positive control, while DMEM medium was used as the negative control. Macrophages were mainly obtained after centrifugation of BALF samples to separate the cells (17). Subsequently, CRISPR/Cas12a components were introduced into macrophages by electroporation and their fluorescence intensity was measured using a microplate reader (**Figure 2D**). Using the clinical diagnosis as the reference standard, a receiver operating characteristic (ROC) curve analysis was employed to determine an optimal signal cutoff value of 3082 for classifying positive results (**Supplementary Figure 1**). The CRISPR/Cas12a assay identified 17 of 18 active TB patients, achieving a sensitivity of 94%, which was significantly higher than culture (67%, 12/18), Xpert (78%, 14/18), TB-DNA (72%, 13/18) and AFB (44%, 7/18). The assay also demonstrated exceptional specificity, correctly identifying all 8 non-TB samples as negative, yielding a specificity of 100% (**Figures 2E, F**, **Table 2**). This was notably higher than the specificity achieved by culture and AFB method. Additionally, a Venn diagram revealed that the CRISPR/Cas12a system detected two TB cases that were missed by both Xpert and culture, further demonstrating its superior sensitivity (**Figure 2G**). Additional performance metrics were calculated for the CRISPR/Cas12a assay in analyzing BALF samples. The positive predictive value (PPV) was 100%, the negative predictive value (NPV) was 91%, and the overall accuracy was 87% (**Table 2**). These findings underscored the system’s high sensitivity, specificity, and accuracy in detecting *Mtb*, where traditional methods such as Xpert and culture were less effective. In summary, the CRISPR/Cas12a assay showed outstanding potential for diagnosing tuberculosis by effectively detecting *Mtb* in RAW264.7 cells and clinical samples. With its enhanced sensitivity, specificity, and accuracy exceeding those of traditional methods, the CRISPR/Cas12a assay stands out as a highly promising tool for improving tuberculosis diagnosis.

**Table 2:** Performance comparison of diagnostic methods for detecting clinical samples.

Methods	Sensitivity (%)	Specificity (%)	PPV (%)	NPV (%)
Culture	67	80	86	57
Xpert	78	100	100	71
TB-DNA	72	100	100	67
AFB	44	80	80	44
CRISPR/Cas12a	94	100	100	91

PPV, positive predictive value; NPV, negative predictive value.

## 4 Conclusion

In summary, the CRISPR/Cas12a assay enabled real-time, *in situ* imaging of intracellular *Mtb* nucleic acids by directly targeting and visualizing *IS6110* DNA in infected macrophages. This assay has demonstrated robust performance in clinical BALF samples, with superior sensitivity, specificity, and accuracy compared to conventional methods such as culture, Xpert, TB-DNA, and AFB staining. These results highlight its potential to transform TB diagnosis. The CRISPR/Cas12a assay for detecting intracellular *Mtb* in living cells holds great potential for biomedical research and clinical diagnostics, particularly in tracking the progression of infectious diseases.

## Data Availability

The raw data supporting the conclusions of this article will be made available by the authors, without undue reservation.
