# Supplementary material for: Ultra-sensitive in situ detection of intracellular Mycobacterium tuberculosis with CRISPR/Cas12a
Source: Front Immunol. 2025 May 21;16:1597654. doi: 10.3389/fimmu.2025.1597654 (PMC12133786; doi:10.3389/fimmu.2025.1597654)
Supplement: Supplementary file 1 [file DataSheet1.docx]

Supplementary Material

# Supplementary Figures


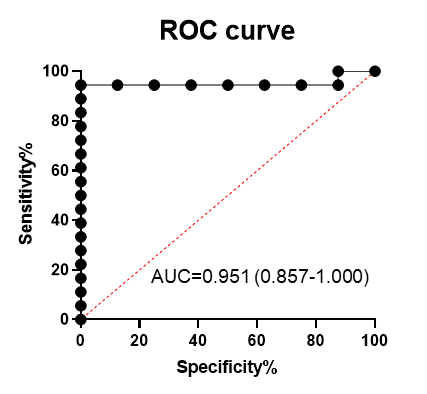


**Supplementary Figure 1.** Diagnostic potential of CRISPR/Cas12a assay was evaluated by ROC (Receiver Operating Characteristic) curve analysis with AUC (Area under curve) within 95% confidence interval (CI).
